# N-Acetyl-Cysteine Supplementation Improves Functional Connectivity Within the Cingulate Cortex in Early Psychosis: A Pilot Study

**DOI:** 10.1093/ijnp/pyz022

**Published:** 2019-07-08

**Authors:** Emeline Mullier, Timo Roine, Alessandra Griffa, Lijing Xin, Philipp S Baumann, Paul Klauser, Martine Cleusix, Raoul Jenni, Yasser Alemàn-Gómez, Rolf Gruetter, Philippe Conus, Kim Q Do, Patric Hagmann

**Affiliations:** 1 Department of Radiology, Lausanne University Hospital (CHUV), Lausanne, Switzerland; 2 Turku Brain and Mind Center, University of Turku, Turku, Finland; 3 Dutch Connectome Lab, Department of Complex Trait Genetics, Center for Neurogenomics and Cognitive Research (CNCR), VU Amsterdam, Amsterdam, The Netherlands; 4 Center for Psychiatric Neuroscience, Department of Psychiatry, Lausanne University Hospital (CHUV), Lausanne, Switzerland; 5 Treatment and Early Intervention in Psychosis Program (TIPP), Service of General Psychiatry, Department of Psychiatry, Lausanne, Switzerland; 6 Laboratory of Functional and Metabolic Imaging, Ecole Polytechnique Fédérale de Lausanne, Lausanne, Switzerland; 7 Medical Image Analysis Laboratory (MIAL), Centre d’Imagerie BioMédicale (CIBM), Lausanne, Switzerland

**Keywords:** N-acetyl-cysteine, early psychosis, functional connectivity, cingulate cortex

## Abstract

**Background:**

There is increasing evidence that redox dysregulation, which can lead to oxidative stress and eventually to impairment of oligodendrocytes and parvalbumin interneurons, may underlie brain connectivity alterations in schizophrenia. Accordingly, we previously reported that levels of brain antioxidant glutathione in the medial prefrontal cortex were positively correlated with increased functional connectivity along the cingulum bundle in healthy controls but not in early psychosis patients. In a recent randomized controlled trial, we observed that 6-month supplementation with a glutathione precursor, N-acetyl-cysteine, increased brain glutathione levels and improved symptomatic expression and processing speed.

**Methods:**

We investigated the effect of N-acetyl-cysteine supplementation on the functional connectivity between regions of the cingulate cortex, which have been linked to positive symptoms and processing speed decline. In this pilot study, we compared structural connectivity and resting-state functional connectivity between early psychosis patients treated with 6-month N-acetyl-cysteine (n = 9) or placebo (n = 11) supplementation with sex- and age-matched healthy control subjects (n = 74).

**Results:**

We observed that 6-month N-acetyl-cysteine supplementation increases functional connectivity along the cingulum and more precisely between the caudal anterior part and the isthmus of the cingulate cortex. These functional changes can be partially explained by an increase of centrality of these regions in the functional brain network.

**Conclusions:**

N-acetyl-cysteine supplementation has a positive effect on functional connectivity within the cingulate cortex in early psychosis patients. To our knowledge, this is the first study suggesting that increased brain glutathione levels via N-acetyl-cysteine supplementation may improve brain functional connectivity.

**Significance Statement**

In clinical trials, N-acetyl-cysteine as an add-on treatment to patients with schizophrenia and early psychosis patients showed significant increase of glutathione brain concentration and improvements in symptomatic expression and processing speed. We here provide a novel insight into the effect of N-acetyl-cysteine supplementation by investigating brain functional connectivity of early psychosis patients. The functional connectivity between the caudal anterior cingulate cortex and the isthmus of cingulate cortex increases after the N-acetyl-cysteine supplementation. This main finding of our study showed that this add-on might be useful for restoring functional connectivity alterations in early psychosis patients, which have been linked to symptoms in previous studies.

## Introduction

Mounting evidence indicates that redox dysregulation and resulting oxidative stress are key players in the pathophysiology of schizophrenia (Steullet et al., 2016). One pathway leading to redox dysregulation in schizophrenia is a deficit in glutathione (GSH), the main antioxidant and redox regulator in the brain, which has been shown to be decreased in some patients’ brain, as directly measured from cerebrospinal fluid or via MR spectroscopy in the frontal lobe (Das et al., 2019; Do et al., 2000). The redox dysregulation model proposes that the combined action of a GSH synthesis deficit of genetic origin and an excess of oxidative stress caused by environmental factors during neurodevelopment, together with neuroinflammation and glutamatergic hypofunction, impairs neural connectivity and synchronization through fast-spiking parvalbumin (GABA) interneuron impairments and deficits in myelination (Do et al., 2009; Hardingham and Do, 2016; Steullet et al., 2016).

In the light of these mechanisms, there has been a great interest in N-acetyl-cysteine (NAC), a GSH precursor and antioxidant. Indeed, the first double-blinded, randomized, placebo-controlled clinical trial by Berk et al. showed that NAC is safe and effective as an add-on to antipsychotic medication to improve negative symptoms and antipsychotic-associated side effects in patients with chronic schizophrenia (Berk et al., 2008). Furthermore, improvements of negative symptoms were replicated in 2 other studies (Farokhnia et al., 2013; Sepehrmanesh et al., 2018). More recently, positive effects of NAC on cognition (Rapado-Castro et al., 2017; Sepehrmanesh et al., 2018) and positive symptoms (Sepehrmanesh et al., 2018) have also been reported.

In a recent randomized controlled trial by our group on the effect of NAC in early psychosis, we reported that a 6-month NAC add-on treatment increased brain GSH levels by 23% in the medial prefrontal cortex (mPFC) of early psychosis patients (EPPs) (Conus et al., 2018). Despite no change in negative symptoms, possibly due to low baseline levels, we observed an improvement in positive symptoms in a subgroup of patients showing high baseline peripheral oxidative status (Conus et al., 2018). In the same trial, we also reported improvement in cognition (particularly, processing speed) (Conus et al., 2018) and low-level auditory processing (Retsa et al., 2018), suggesting a more general effect of NAC intake and GSH levels on signal processing and sensory integration. Accordingly, in patients with the NAC add-on treatment, we also observed an improvement in white matter diffusion properties in the fornix. The white matter changes correlated with the augmentation of brain GSH levels, suggesting a possible restorative process along the fornix bundle (Klauser et al., 2018).

Taken together, these findings indicate that NAC supplementation can increase brain GSH levels and improve symptoms and processing speed in EPPs, possibly due to underlying changes in white matter diffusion properties. Indeed, white matter diffusion properties and synchronizations are disrupted in schizophrenia as reported in structural (Camchong et al., 2011; Whitford et al., 2014; Griffa et al., 2015; Klauser et al., 2017) and functional (Calhoun et al., 2009; Camchong et al., 2011) connectivity studies in both chronic schizophrenia and EPPs (Alonso-Solis et al., 2012; Zhang et al., 2014; Li et al., 2017). The most consistent brain network alterations evolve in a rostro-caudal fashion, from frontal regions at early stages, to a more widespread dysconnectivity involving all cerebral lobes and including the cerebellum in long-term schizophrenia (Pettersson-Yeo et al., 2011; Fornito et al., 2012; Van Den Heuvel and Fornito, 2014; Bartholomeusz et al., 2017; Skåtun et al., 2017). Some studies showed that these alterations correlate with symptom severity (Camchong et al., 2011; Whitford et al., 2014; Li et al., 2017).

Furthermore, functional and structural connectivity values in the cingulate correlate with mPFC GSH levels in healthy controls. In EPPs, only the association between GSH levels and structural connectivity values is preserved, whereas the association between GSH and functional connectivity is disrupted (Monin et al., 2015). These findings suggest that brain connectivity features in the cingulate may be critically related to brain GSH levels measured in the mPFC and that this association may be altered early in the time course of psychosis.

Growing evidence shows that NAC can alleviate several symptomatic dimensions of psychosis (Berk et al., 2008; Lavoie et al., 2008; Carmeli et al., 2012; Shungu, 2012; Farokhnia et al., 2013), and it can restore changes in white matter diffusion properties. However, the effect of NAC intake on functional connectivity alterations in early psychosis has not yet been tested. In the current study, we investigated whether 6-month NAC supplementation can restore functional connectivity along the cingulum bundle. There were 3 reasons for focusing on the cingulum. First, the cingulum bundle is a key region implicated in the pathophysiology of schizophrenia and whose structural integrity has been linked with positive symptoms (Whitford et al., 2014; Kates et al., 2015) and impairment of processing speed (Karbasforoushan et al., 2015; Kochunov et al., 2017), 2 symptomatic dimensions of which were improved in the study by Conus and colleagues (Conus et al., 2018). Second, we previously observed a correlation between brain GSH levels and functional connectivity along the cingulum in healthy individuals, indicating that higher GSH levels correspond to better synchronization of the medial prefrontal and posterior cingulate cortices. This association was disturbed in EPPs (Monin et al., 2015). Lastly, we recently showed that NAC has the ability to increase GSH levels in the mPFC, highlighting again that the cingulate is an important region to focus on. The current study is based on a subgroup of the cohort investigated in (Conus et al., 2018), which was composed of patients who agreed to participate in a very demanding imaging study. Given the increase in GSH levels in the mPFC in this same cohort, we hypothesized that NAC supplementation would lead to an increase in functional and structural connectivity along the cingulum bundle, which would correlate with changes in brain GSH levels.

## Materials and Methods

### Clinical Trial Protocol

NAC (2700 mg/d) and placebo were administered to EPPs for 6 months following a double-blinded randomized design. Resting-state functional magnetic resonance imaging (fMRI), diffusion spectrum imaging (DSI), and magnetic resonance spectroscopy (MRS) were performed at baseline and at the end of the study after 6 months of NAC administration (Swiss Medic [2008DR2308], ClinicalTrial.gov [NCT01354132]). The study reported here is based on a subsample of a larger clinical trial (Conus et al., 2018).

### MRI Study Participants

Participants were recruited from the Treatment and Early Intervention in Psychosis Program (Lausanne University Hospital, Switzerland) (Baumann et al., 2013). Patients meeting criteria for early psychosis were included in the trial. The Lausanne Psychosis cohort was created under the supervision of ethics boards with representatives at different levels (CHUV/Canton/Fédéral). The study was approved by the local research ethic committee (Commission cantonale d’éthique de la recherche sur l’être humain), and written informed consent was obtained from all participants. A complete description of the study and of the cohort can be found elsewhere (Conus et al., 2018). Among the 63 randomized participants of this study (32 for the NAC arm and 31 for the placebo arm), 20 patients (9 NAC, 11 placebo) agreed to participate in a complementary MRI study. These 20 patients (7 women, 13 men; 15 right-handed, 4 left-handed, 1 ambidextrous; aged 25 ± 6 years) were scanned at baseline and at the 6-month follow-up after NAC or placebo add-on. A total of 74 age and gender-matched healthy controls (27 women, 47 men; 62 right-handed, 9 left-handed, 2 ambidextrous; aged 26 ± 6 years) were recruited and assessed by the Diagnostic Interview for Genetic Studies (Preisig et al., 1999). Major mood, psychotic or substance-use disorders, and having a first-degree relative with a psychotic disorder were exclusion criteria for the healthy subjects. Neurological disorders and severe head trauma were exclusion criteria for all subjects.

### Structural, Diffusion, and Functional MRI Acquisitions

T1-weighted volumes and fMRI recordings were acquired on a 3-Tesla scanner (Trio, Siemens Medical, Germany) equipped with a 32-channel head coil. For the T1-weighted volumes, a magnetization-prepared rapid acquisition gradient echo sequence was acquired with 1-mm in-plane resolution and 1.2-mm slice thickness, covering 240 ± 257 ± 160 voxels. The TR, TE, and TI were 2300, 2.98, and 900 ms, respectively. For the fMRI recordings, each subject was scanned in resting-state conditions using a standard gradient echo planar imaging sequence sensitive to blood oxygen-level-dependent contrast. An axial plane was used with a 64- ± 58-voxel matrix (resolution 3.3 ± 3.3 mm^3^). Thirty-two slices of 3.3-mm thickness with a 0.3-mm gap were acquired. Acquisition and repetition times were 9 minutes and 1920 milliseconds, respectively. A DSI sequence was also acquired during the same MRI session with a 2.2- ± 2.2- ± 3-mm^3^ resolution, covering 96 ± 96 ± 34 voxels. The TR and TE were 6100 and 144 ms, respectively. A q4-half acquisition scheme was used with a maximum b-value of 8000 s/mm^2^ and 1 b_o_ volume (Wedeen et al., 2008). The same MRI protocols were applied to both the EPP and control groups.

### MRS Acquisitions

The MRS experiment was performed with a transverse electromagnetic volume head coil (MR Instruments, Minneapolis, MN). The magnetic field homogeneity was optimized by adjusting first- and second-order shims using FAST(EST)MAP (Gruetter, 1993). In vivo proton nuclear magnetic resonance spectra were acquired from a volume of interest positioned in the medial bilateral prefrontal lobe using the short-TE spin-echo full-intensity acquired localized single voxel spectroscopy technique (Mekle et al., 2009). The scan covered a volume of interest of 20 ± 25 ± 25 mm^3^ with TR/TE = 4000/6 ms, an acquisition bandwidth of 2 kHz, an averaged number of 148, and a vector size of 2048. The GSH concentration was quantified via the water-suppressed in vivo proton nuclear magnetic resonance imaging resonance spectra using LCModel (Provencher, 1993) with the unsuppressed water proton nuclear magnetic resonance spectra as an internal reference. The spectral range for analysis was set to 0.2 to 4.2 ppm. GSH was quantified with a Cramer-Rao lower bounds of 10 ± 3%. More details can be found in Xin and al. (Xin et al., 2016).

### Data Processing

Magnetization-prepared rapid acquisition gradient echo volumes were segmented into white matter, gray matter, and cerebrospinal fluid compartments. The gray matter volume was parcellated into 68 cortical and 14 subcortical anatomical regions and the brainstem according to the Desikan-Killiany atlas (Desikan et al., 2006) using FreeSurfer software (Fischl et al., 2002) (https://surfer.nmr.mgh.harvard.edu/).

Whole-brain tractography was performed on reconstructed DSI data using CMTK software (Daducci et al., 2012) (http://connectomics.org/), which allowed individual structural connectivity matrices to be estimated. The number of streamlines connecting each pair of gray matter regions was used as a measure of structural connectivity strength. Furthermore, we built a group-representative structural connectome by averaging the individual structural connectivity matrices over the 74 healthy subjects. Structural connections that were not present in all the healthy subjects were discarded from further analyses.

fMRI data were processed according to a state-of-the-art pipeline including discarding the first 5 time points for signal stabilization, slice-timing correction, motion correction, regressions of 6 motion parameters, averaging white matter and cerebrospinal fluid signals, linear detrending, and bandpass filtering (0.01–0.1 Hz) using CMTK software. The structural volumes were linearly registered to the individual fMRI spaces. Voxelwise fMRI time series were averaged over the 83 gray matter regions. Functional connectivity was assessed by computing the Pearson’s correlation coefficient between the temporal signals of brain region pairs. Negative values were not discarded, but absolute values were instead taken. Indeed, recent studies confirm that negative correlation values may have biological origins (Chai et al., 2012) and present specific features in schizophrenia (Parente et al., 2018). We filtered the functional connections using the group-representative structural connectome. We performed the filtering to discard the weakest functional connections (Goñi et al., 2014) and reduce the number of false positives without introducing a threshold bias (Drakesmith et al., 2015; van den Heuvel et al., 2017).

### Connectivity Analysis

**Figure 1.**
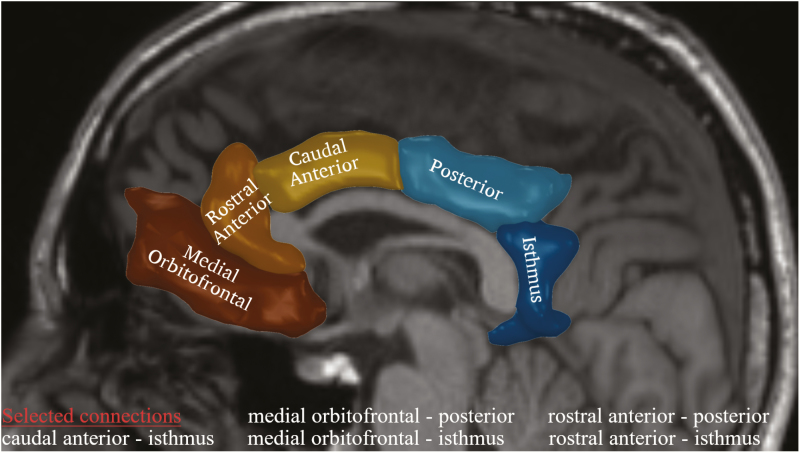
Five regions along the cingulum bundle and selected connections for the analysis. Changes occurring between the frontal (medial orbito frontal, rostral anterior, and caudal anterior) and posterior (posterior and isthmus) cingulate regions were investigated. The connection between the posterior cingulate and caudal anterior cingulate was excluded considering the proximity of the 2 regions.

The resting-state functional network of each subject was defined using the functional connectivity matrix, with the 83 brain regions corresponding to the 83 nodes of the network and the pairwise Pearson’s correlation values representing the edges’ strength. The structural network was defined using the number of streamlines as the edge weight. As a first step, we investigated the effect of NAC or placebo intake on the functional and structural connectivity of the cingulate cortex, which have been shown to be impaired in schizophrenia (Van Den Heuvel and Fornito, 2014; Whitford et al., 2014; Monin et al., 2015). More specifically, we considered the functional and structural connectivity between 3 anterior (medial orbitofrontal, rostral anterior cingulate, and caudal anterior cingulate cortices) and 2 posterior (posterior cingulate and isthmus of cingulate cortices) regions of the cingulate cortex (Figure 1), resulting in 6 connections to be tested. The connectivity values of the left and right hemispheres were averaged. We compared the baseline connectivity values with the 6-month connectivity values for both the NAC and placebo EPP groups. Furthermore, we compared the brain connectivity of each EPP group with that of the control subjects. Next, we characterized integration and segregation properties of the functional and structural brain networks using graph theoretical measures. At the global level, we computed the global efficiency and average clustering coefficient of each individual (structural and functional) brain network. The global efficiency represents the efficiency of communication through shortest paths in the brain network, while the average clustering coefficient quantifies the level of local connectedness in the network. At the local level, we computed the node betweenness centrality and the edge betweenness centrality for the regions and connections of the cingulate cortex (Figure 1). The betweenness centrality quantifies the number of shortest paths in the network passing through a given node or connection. Here, the betweenness centrality quantifies the centrality of the cingulate cortex regions and cingulum connections within the overall brain network. The graph measures were computed with the MATLAB Brain Connectivity Toolbox (Rubinov and Sporns, 2010). The formal definitions of these measures can be found in (Rubinov and Sporns, 2010).

### Statistical Analysis

The relationship between the brain connectivity values and the GSH levels was assessed with the Spearman correlation coefficient. Statistical analyses were performed using MATLAB functions (MATLAB R2017a) and in-house code.

## Results

**Figure 2.**
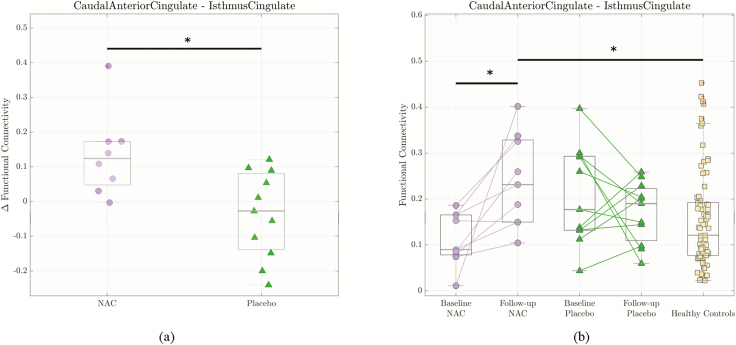
Functional connectivity changes for caudal anterior cingulate cortex-isthmus of cingulate cortex connection. The central grey line represents the median of the distribution. The edges of the box are the 25th and 75th percentiles (*P < .05). (a) The median of the functional connectivity differences before and after supplementation (ΔFC) was significantly higher for the N-acetyl-cysteine (NAC) arm and placebo arm (1-sided Wilcoxon ranksum test, *P* = .006, *P* = .03 adjusted with Bonferroni correction). (b) The median of functional connectivity values for the NAC group at follow-up was significantly higher than for the NAC group at baseline (1-sided Wilcoxon signed-rank test, *P *= .0039) and healthy controls (1-sided Wilcoxon ranksum test, *P *= .0088).

First, we investigated the effect of NAC on functional connectivity in the cingulate cortex. To do this, we analyzed the relative variation of functional connectivity values between the anterior and posterior cingulate regions before and after the supplementation with NAC or placebo. The median of functional connectivity differences before and after supplementation (ΔFC) was significantly higher in NAC compared with placebo patients for the caudal anterior cingulate-isthmus of cingulate connection (1-sided Wilcoxon: *P *=* .*006, *P *=* .*03 adjusted with Bonferroni correction) (Figure 2a). There were no significant changes for the other connections between the anterior and posterior parts of the cingulate cortex. For this reason, we focused our further analyses on characterizing the functional connectivity changes between the caudal anterior cingulate and isthmus of cingulate cortices.

In a second step, we extended our investigation to possible differences in functional connectivity between the caudal anterior cingulate and isthmus of cingulate cortices in patients compared with the healthy controls. The distributions of functional connectivity values for both patient’s baseline and follow-up data were calculated. Post-hoc tests were performed to compare the NAC and placebo groups with a control group. No significant changes were found between EPPs at baseline and healthy controls (one-sided Wilcoxon *P* = .27). We observed a heterogeneity in the 2 EPPs baseline groups, NAC and placebo, which was not significant (Wilcoxon *P* = .068). However, the results showed a significant increase in functional connectivity at the follow-up compared with baseline for the NAC patients (1-sided paired Wilcoxon, *P* = .0039), whereas no change was observed for the placebo patients (1-sided paired Wilcoxon *P* = .41), as expected from the results of Figure 2A. Moreover, the functional connectivity between the caudal and isthmus of the cingulate cortices in the NAC group after 6-month supplementation was larger than that of the control group (1-sided Wilcoxon, *P* = .0088) (Figure 2b).

**Figure 3.**
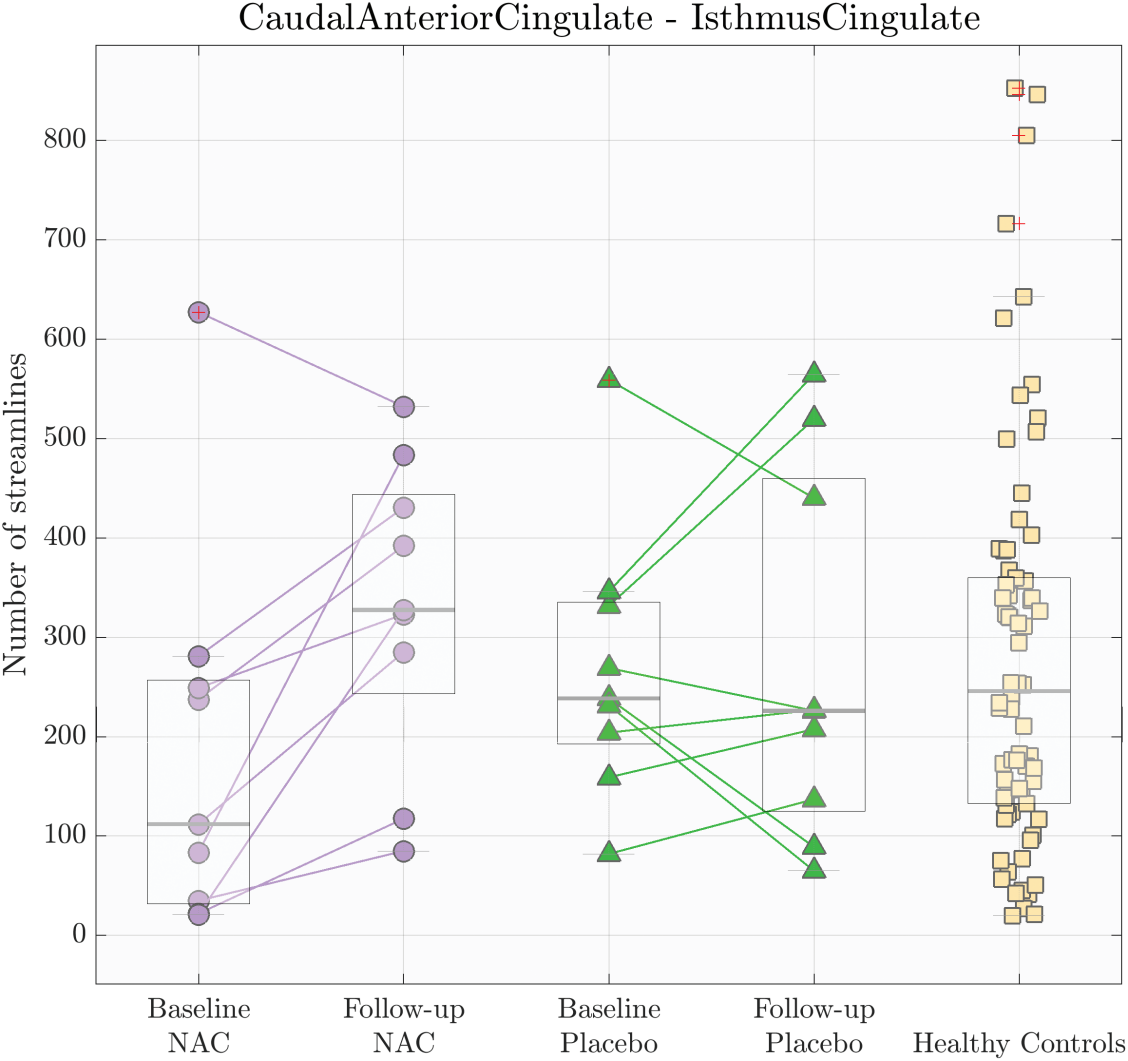
Structural connectivity of connection between the caudal anterior cingulate and isthmus of cingulate cortices. The central grey line represents the median of the distribution. The edges of the box are the 25th and 75th percentiles (*P < .05). The median of number of streamlines had a trend to increase for N-acetyl-cysteine (NAC) group compared with placebo and healthy control groups after 6-month supplementation (1-sided Wilcoxon ranksum test between NAC at baseline and NAC at follow-up [*P = .*082] and between NAC at follow-up and healthy controls [*P = .*14]).

Similarly, we explored possible structural connectivity alterations between the anterior and the posterior parts of the cingulate cortex (5 connections of interest; Figure 1); as such we compared the NAC, placebo, and healthy control groups. We used the number of streamlines as an edge weight to characterize the structural connectivity. No significant differences were found after correction for multiple comparisons. Nevertheless, in view of the findings regarding functional connectivity impairments, we focused our analysis on the caudal-isthmus connection. After 6-month supplementation of NAC, this connection showed a trend of an increased number of streamlines connecting the caudal and isthmus of the cingulate cortices (1-sided paired Wilcoxon, *P *= .082, *P *= .41 adjusted with Bonferroni correction) (Figure 3).

**Figure 4.**
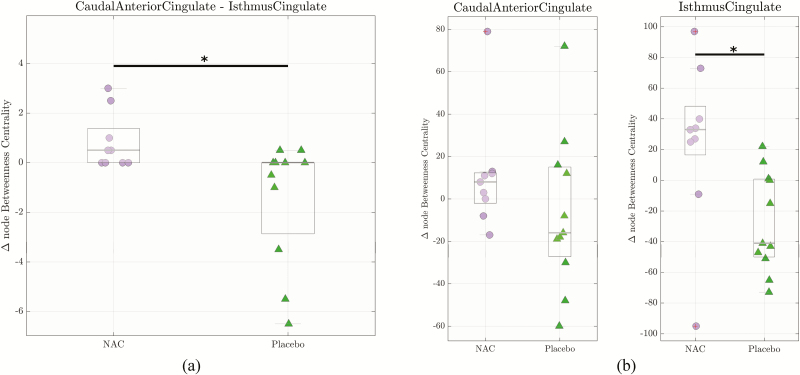
Edge and node betweenness centrality for caudal anterior cingulate cortex-isthmus of cingulate cortex connection. The central grey line represents the median of the distribution. The edges of the box are the 25th and 75th percentiles (*P < .05). (a) The median of Δ edge betweenness centrality was significantly higher for N-acetyl-cysteine (NAC) arm than placebo arm (1-sided Wilcoxon ranksun test, *P *= .0073). (b) The median of Δ node betweenness centrality values in the isthmus of cingulate cortex is significantly increased for the NAC arm compared with the placebo arm (1-sided Wilcoxon ranksum test, *P *= .0049). The same trend was found in the caudal anterior cingulate cortex (Wilcoxon ranksum test, *P *= .11 for average left and right hemisphere, *P *= .01 for right hemisphere only).

We hypothesized that the increase under NAC supplementation of the functional connectivity between the caudal cingulate and isthmus of the cingulate cortices could imply a reorganization of the brain functional network around this specific connection. First, we characterized the global topological changes of the brain functional networks by computing the global efficiency and average clustering coefficient of the network. No significant changes were found between the baseline and follow-up data for these global measures. Second, we assessed the nodal betweenness centrality of the caudal cingulate and isthmus of cingulate regions, and the edge betweenness centrality of their functional connection. In regard to these local network measures, the change (between baseline and 6-month follow-up) in edge between centrality was significantly larger in NAC patients than in placebo patients (1-sided Wilcoxon, *P *= .0073) (Figure 4a). Moreover, the change in nodal betweenness centrality for the isthmus of cingulate region was significantly higher for NAC patients than for placebo patients (1-sided Wilcoxon, *P *= .0049) (Figure 4b). The same trend was found for the caudal anterior cingulate region (1-sided Wilcoxon, *P *= .11 for average left-right hemispheres, significant in the right hemisphere, *P *= .01).

**Figure 5.**
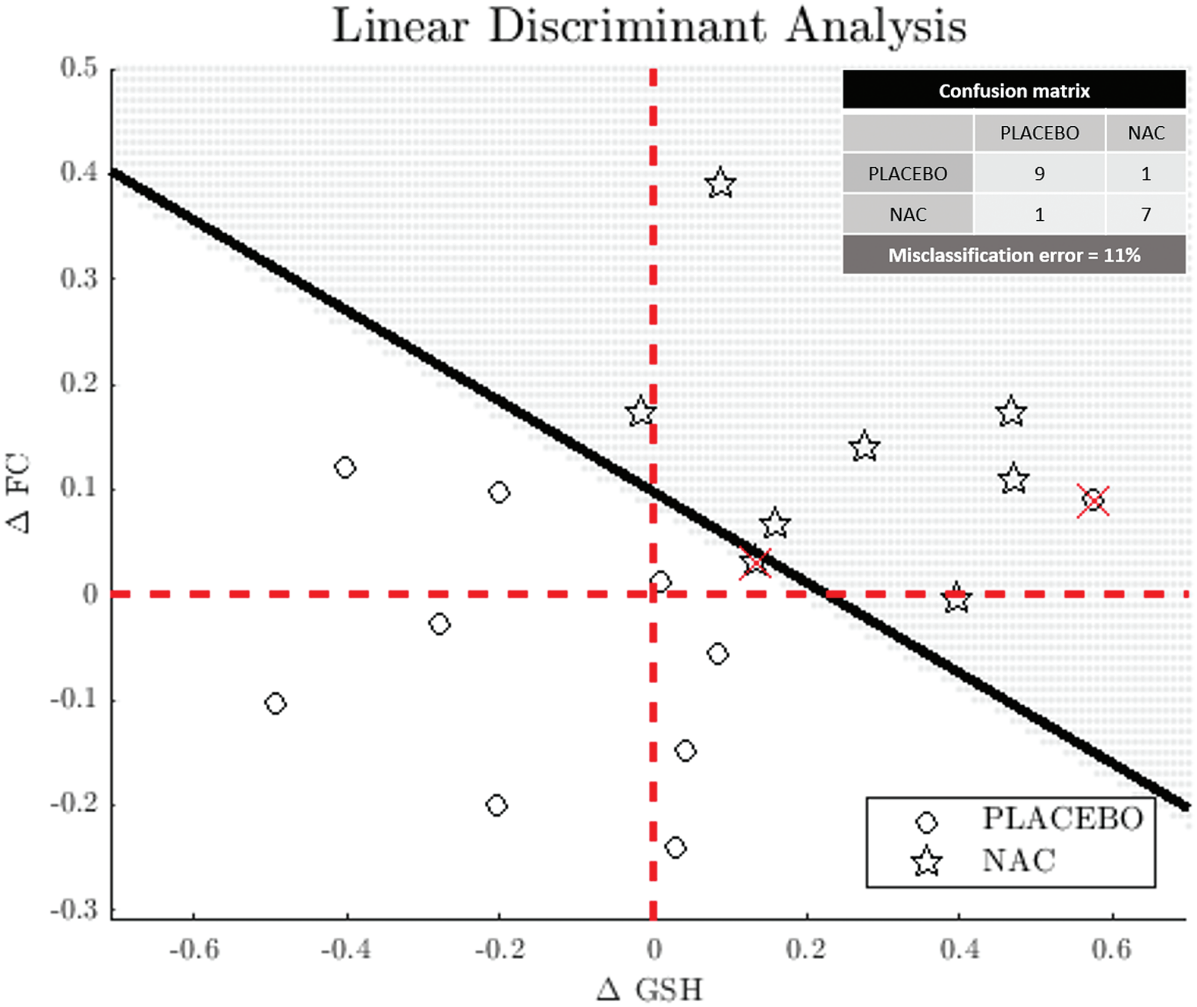
Linear discriminant analysis. Δ Glutathione (GSH) and median of the functional connectivity differences before and after supplementation (ΔFC) are used as features of a linear discriminant analysis to classify the subjects into the 2 arms. The misclassification error was estimated at 5% via a resubstitution validation and 11% using a k-fold cross-validation procedure.

On one hand, our results showed an increase of functional connectivity in the cingulate regions. On the other hand, Conus and al. (Conus et al., 2018) already showed in the larger cohort of this clinical trial a significant increase of GSH brain concentration, suggesting that the supplementation of NAC, precursor of GSH, was able to help restore the redox dysregulation mechanism responsible for connectivity alteration in schizophrenia (Steullet et al., 2016). To verify this hypothesis, we also investigated the relationship between ΔFC and GSH changes (ΔGSH) in NAC patients. The correlation between ΔFC and ΔGSH (Spearman’s correlation coefficient, r = 0.33, *P *= .17) was not significant. Then, a linear discriminant analysis was performed on the (ΔFC, ΔGSH) values with respect to the 2 groups of NAC patients and placebo patients (Figure 5). The classification error on our dataset was 11%. We estimated the expected classification error on a new dataset at 11% using a k-fold cross-validation procedure based on the resampling of our data.

## Discussion

The main finding of this study is that functional connectivity along the cingulum bundle was increased after 6-month NAC supplementation in EPPs. We observed that functional connectivity between the caudal anterior cingulate and isthmus of cingulate cortices increased with NAC supplementation. We further hypothesized that baseline functional connectivity was decreased between EPPs compared with healthy controls along the cingulum bundle. However, no significant differences in functional connectivity were found between the 2 groups, which may be due to the effect of the disease in early psychosis not being strong enough or the sample being too small. The functional connectivity increase after NAC supplementation might be related to compensatory mechanisms, considering that functional connectivity loss in the cingulate regions have been previously observed in patients with psychosis (Van Den Heuvel and Fornito, 2014; Skåtun et al., 2017b). Changes in functional connectivity after NAC intake has been studied in the context of smoking cessation (Froeliger et al., 2015), where NAC restores fronto-striatal resting-state functional connectivity in preventing relapse during smoking cessation. In summary, this is the first study examining the impact of NAC on functional connectivity in EPPs. In spite of a heterogeneity between our 2 EPPs groups at baseline, the relative difference of functional connectivity between the anterior and posterior regions of the cingulate cortex was strengthened in the NAC group after 6 months of treatment compared with the placebo group and baseline values in healthy controls.

Changes in functional connectivity are difficult to interpret quantitatively because of the complex physiology of the BOLD signal (Gauthier and Fan, 2019). However, evidence relates the BOLD signal to the electrophysiological brain activity (Logothetis and Wandell, 2004) and suggests that an increase in FC could reflect higher synchronization between the corresponding brain regions. This higher synchronization could have different origins and could be related to, for instance, the reorganization of functional brain networks due to white matter alterations.

We thus provide evidence that the functional connections along the cingulum may be rearranged by NAC supplementation. Indeed, edge betweenness centrality was significantly increased for the same caudal-isthmus of cingulate connection. A higher number of shortest paths passed through this connection after NAC supplementation, suggesting that this connection becomes more efficient in linking rostral to caudal regions of the cingulate cortex. This functional network reorganization could be a local compensatory process to alleviate the overall connectivity disruptions reported in the literature by exploiting new communication pathways and could reflect underlying white matter changes.

Functional connectivity abnormalities in schizophrenia may be underpinned by white matter alterations (Zalesky et al., 2011; Klauser et al., 2017). Thus, we next examined whether changes in structural connectivity may underlie changes in functional connectivity. Indeed, a restoration of structural alterations could explain improvements in functional connectivity by rehabilitating the structural pathways that convey the transmission of information. Even if the relationship between structural and functional connectivity is not yet fully understood, they are strongly related and functional connectivity to some extent reflects the underlying structural scaffold (Honey et al., 2010; Cabral et al., 2013; Goñi et al., 2014). In our sample, the structural connectivity (as quantified by the number of streamlines) between the caudal and isthmus of cingulate regions was increased to a trend level after 6-month NAC supplementation. A longer period of molecule supplementation or a larger cohort might be necessary to detect statistically significant changes in the white matter organization. In this regard, it is interesting to note that in the same cohort, 6-month NAC supplementation improved the white matter properties of the body of the fornix (as assessed with diffusion MRI and generalized fractional anisotropy) (Klauser et al., 2018). This white matter region is much thinner than the cingulum and has been shown to be very vulnerable to oxidative stress (Corcoba et al., 2015).

One of the possible mechanisms for explaining the observed functional connectivity alterations in schizophrenia is the hypothesis that a redox dysregulation along with neuroinflammation and glutamatergetic system NMDAR hypofunction would cause macro- and microcircuit impairments resulting in altered functional connectivity (Steullet et al., 2016). To explore this hypothesis, we investigated the relationship between the brain GSH concentration and the functional connectivity changes observed in our sample. However, the direct relationship between GSH levels and functional connectivity values between the caudal and isthmus cortices was not significant. Nevertheless, we demonstrated a dependence between these parameters by using linear discriminant analysis to predict a subject’s alliance to the NAC or placebo groups. Indeed, ΔFC and ΔGSH values allowed for accurate classification of the patients into the 2 groups. Replication of these results in a larger cohort could reinforce the evidence for the redox dysregulation model. Moreover, cingulate functional connectivity values and GSH levels may be useful markers in monitoring the NAC treatment response as these 2 markers have already been related to symptoms and cognitive scores (Yan et al., 2012; Conus et al., 2018).

Our study focused only on the connectivity within the cingulate cortex. Indeed, the human cingulate cortex is a functionally heterogeneous structure (Margulies et al., 2007), which can be subdivided into regions with specific functional and neurobiological profiles (Yu et al., 2011). Moreover, diffusion properties of the cingulate subdivisions allow for differentiation of the uniform cingulum white matter tract into more specific tracts (Jones et al., 2013). In schizophrenia, functional connectivity between these cingulate subdivisions and the other brain regions is significantly altered (Wang et al., 2015). However, the nature of the alteration is highly dependent on the fMRI signal processing steps, particularly the regression of the global signal (Wang et al., 2015). Similarly, aberrant functional connectivity has been found in schizophrenia patients between the default mode network, notably involving the medial orbitofrontal cortex, the posterior cingulate cortex, and/or some regions of the cingulate cortex and other functional systems. These aberrations describe either a hyperconnectivity of the default mode network (Whitfield-Gabrieli et al., 2009) or a mix of hyper- and hypoconnectivity dependent on these regions (Yan et al., 2012). However, the connectivity within the cingulate cortex, as studied in our analysis, is sparsely studied. The functional connectivity within the default mode network tends to be lower in patients with schizophrenia compared with healthy populations (Bastos-Leite et al., 2015; Du et al., 2016). The changes of functional connectivity within the cingulate cortex observed after NAC supplementation showed local modification within the cingulate subnetwork, but the global effect on the whole brain remains to be studied in a larger cohort. Further analyses could explore how the alterations of functional connectivity within the cingulate subnetwork may impact the connectivity between other brain subnetworks and connectivity of other functional systems.

Betweenness centrality values are highly dependent on the reliability of the functional network estimation. One of the main limitations in this estimation is to find a balance between sensitivity and specificity by choosing a threshold for the connectivity values (Zalesky et al., 2016). An excess of false positive or false negative connections could significantly affect a range of network measures, such as the global efficiency and clustering coefficient. However, the application of a threshold can result in artifactual differences between the average degree and the number of nodes of the different networks, thus changing the essential conditions for network comparison (van Wijk et al., 2010). The application of a proportional threshold could reduce this problem by maintaining the same density over the different networks. However, fixing the density of the network could increase the number of spurious edges in pathological networks suffering from connectivity alterations and could bias the comparison between the different arms (van den Heuvel et al., 2017; Hallquist and Hillary, 2019). In our analysis, we filtered our functional connectivity matrix by the structural scaffold without applying any threshold. This method has been developed based on the strong structure-function relationship and is designed to reduce the number of false positive connections in the functional network.

The limitations of the current study deserve some further considerations. First, the small sample size of our study limits the generalizability of the findings, which should be replicated in a larger sample. Second, the nature of the connectivity alterations in fMRI studies depend on the functional signal processing pipeline and notably the regression of the global signal. A larger cohort and a comparison of different signal processing steps would allow for a deeper exploration of the effect of NAC on connectivity of functional brain networks.

In conclusion, we investigated the connectivity changes within the cingulate cortex of EPPs after NAC supplementation. We found a significant change in functional connectivity between the caudal anterior cingulate cortex and the isthmus of cingulate cortex after NAC supplementation. Further studies are needed to assess to what extent NAC may be useful for restoring functional connectivity in the cingulate cortex in early psychosis.

## References

[CIT0001] Alonso-Solís A, Corripio I, de Castro-Manglano P, Duran-Sindreu S, Garcia-Garcia M, Proal E, Nuñez-Marín F, Soutullo C, Alvarez E, Gómez-Ansón B, Kelly C, Castellanos FX (2012) Altered default network resting state functional connectivity in patients with a first episode of psychosis. Schizophr Res 139:13–18. 22633527 10.1016/j.schres.2012.05.005 PMC3393844

[CIT0002] Bartholomeusz CF, Cropley VL, Wannan C, Di Biase M, McGorry PD, Pantelis C (2017) Structural neuroimaging across early-stage psychosis: aberrations in neurobiological trajectories and implications for the staging model. Aust N Z J Psychiatry 51:455–476. 27733710 10.1177/0004867416670522

[CIT0003] Bastos-Leite AJ, Ridgway GR, Silveira C, Norton A, Reis S, Friston KJ (2015) Dysconnectivity within the default mode in first-episode schizophrenia: a stochastic dynamic causal modeling study with functional magnetic resonance imaging. Schizophr Bull 41:144–153. 24939881 10.1093/schbul/sbu080 PMC4266292

[CIT0004] Baumann PS, Crespi S, Marion-Veyron R, Solida A, Thonney J, Favrod J, Bonsack C, Do KQ, Conus P (2013) Treatment and early intervention in psychosis program (TIPP-lausanne): implementation of an early intervention programme for psychosis in Switzerland. Early Interv Psychiatry 7:322–328. 23445318 10.1111/eip.12037

[CIT0005] Berk M, Copolov D, Dean O, Lu K, Jeavons S, Schapkaitz I, Anderson-Hunt M, Judd F, Katz F, Katz P, Ording-Jespersen S, Little J, Conus P, Cuenod M, Do KQ, Bush AI (2008) N-acetyl cysteine as a glutathione precursor for schizophrenia–a double-blind, randomized, placebo-controlled trial. Biol Psychiatry 64:361–368. 18436195 10.1016/j.biopsych.2008.03.004

[CIT0006] Cabral J, Fernandes HM, Van Hartevelt TJ, James AC, Kringelbach ML, Deco G (2013) Structural connectivity in schizophrenia and its impact on the dynamics of spontaneous functional networks. Chaos 23:046111. 24387590 10.1063/1.4851117

[CIT0007] Calhoun VD, Eichele T, Pearlson G (2009) Functional brain networks in schizophrenia: a review. Front Hum Neurosci 3:17. 19738925 10.3389/neuro.09.017.2009 PMC2737438

[CIT0008] Camchong J, MacDonald AW 3rd, Bell C, Mueller BA, Lim KO (2011) Altered functional and anatomical connectivity in schizophrenia. Schizophr Bull 37:640–650. 19920062 10.1093/schbul/sbp131 PMC3080691

[CIT0009] Carmeli C, Knyazeva MG, Cuénod M, Do KQ (2012) Glutathione precursor N-acetyl-cysteine modulates EEG synchronization in schizophrenia patients: a double-blind, randomized, placebo-controlled trial. Plos One 7:e29341. 22383949 10.1371/journal.pone.0029341 PMC3285150

[CIT0010] Chai XJ, Castañón AN, Ongür D, Whitfield-Gabrieli S (2012) Anticorrelations in resting state networks without global signal regression. Neuroimage 59:1420–1428. 21889994 10.1016/j.neuroimage.2011.08.048 PMC3230748

[CIT0011] Conus P, et al (2018) N-acetylcysteine in a double-blind randomized placebo-controlled trial: toward biomarker-guided treatment in early psychosis. Schizophr Bull 44:317-327. 10.1093/schbul/sbx093 PMC5815074 29462456

[CIT0012] Corcoba A, Steullet P, Duarte JM, Van de Looij Y, Monin A, Cuenod M, Gruetter R, Do KQ (2015) Glutathione deficit affects the integrity and function of the fimbria/fornix and anterior commissure in mice: relevance for schizophrenia. Int J Neuropsychopharmacol 19:pyv110. 26433393 10.1093/ijnp/pyv110 PMC4815475

[CIT0013] Daducci A, Gerhard S, Griffa A, Lemkaddem A, Cammoun L, Gigandet X, Meuli R, Hagmann P, Thiran JP (2012) The connectome mapper: an open-source processing pipeline to map connectomes with MRI. PLoS One 7:e48121. 23272041 10.1371/journal.pone.0048121 PMC3525592

[CIT0014] Das TK, Javadzadeh A, Dey A, Sabesan P, Théberge J, Radua J, Palaniyappan L (2019) Antioxidant defense in schizophrenia and bipolar disorder: a meta-analysis of MRS studies of anterior cingulate glutathione. Prog Neuropsychopharmacol Biol Psychiatry 91:94–102. 30125624 10.1016/j.pnpbp.2018.08.006

[CIT0015] Desikan RS, Ségonne F, Fischl B, Quinn BT, Dickerson BC, Blacker D, Buckner RL, Dale AM, Maguire RP, Hyman BT, Albert MS, Killiany RJ (2006) An automated labeling system for subdividing the human cerebral cortex on MRI scans into gyral based regions of interest. Neuroimage 31:968–980. 16530430 10.1016/j.neuroimage.2006.01.021

[CIT0016] Do KQ, Trabesinger AH, Kirsten-Krüger M, Lauer CJ, Dydak U, Hell D, Holsboer F, Boesiger P, Cuénod M (2000) Schizophrenia: glutathione deficit in cerebrospinal fluid and prefrontal cortex in vivo. Eur J Neurosci 12:3721–3728. 11029642 10.1046/j.1460-9568.2000.00229.x

[CIT0017] Do KQ, Cabungcal JH, Frank A, Steullet P, Cuenod M (2009) Redox dysregulation, neurodevelopment, and schizophrenia. Curr Opin Neurobiol 19:220–230. 19481443 10.1016/j.conb.2009.05.001

[CIT0018] Drakesmith M, Caeyenberghs K, Dutt A, Lewis G, David AS, Jones DK (2015) Overcoming the effects of false positives and threshold bias in graph theoretical analyses of neuroimaging data. Neuroimage 118:313–333. 25982515 10.1016/j.neuroimage.2015.05.011 PMC4558463

[CIT0019] Du Y, Pearlson GD, Yu Q, He H, Lin D, Sui J, Wu L, Calhoun VD (2016) Interaction among subsystems within default mode network diminished in schizophrenia patients: a dynamic connectivity approach. Schizophr Res 170:55–65. 26654933 10.1016/j.schres.2015.11.021 PMC4707124

[CIT0020] Farokhnia M, Azarkolah A, Adinehfar F, Khodaie-Ardakani MR, Hosseini SM, Yekehtaz H, Tabrizi M, Rezaei F, Salehi B, Sadeghi SM, Moghadam M, Gharibi F, Mirshafiee O, Akhondzadeh S (2013) N-acetylcysteine as an adjunct to risperidone for treatment of negative symptoms in patients with chronic schizophrenia: a randomized, double-blind, placebo-controlled study. Clin Neuropharmacol 36:185–192. 24201233 10.1097/WNF.0000000000000001

[CIT0021] Fischl B, Salat DH, Busa E, Albert M, Dieterich M, Haselgrove C, van der Kouwe A, Killiany R, Kennedy D, Klaveness S, Montillo A, Makris N, Rosen B, Dale AM (2002) Whole brain segmentation: automated labeling of neuroanatomical structures in the human brain. Neuron 33:341–355. 11832223 10.1016/s0896-6273(02)00569-x

[CIT0022] Fornito A, Zalesky A, Pantelis C, Bullmore ET (2012) Schizophrenia, neuroimaging and connectomics. Neuroimage 62:2296–2314. 22387165 10.1016/j.neuroimage.2011.12.090

[CIT0023] Froeliger B, McConnell PA, Stankeviciute N, McClure EA, Kalivas PW, Gray KM (2015) The effects of N-acetylcysteine on frontostriatal resting-state functional connectivity, withdrawal symptoms and smoking abstinence: a double-blind, placebo-controlled fmri pilot study. Drug Alcohol Depend 156:234–242. 26454838 10.1016/j.drugalcdep.2015.09.021 PMC4633320

[CIT0024] Gauthier CJ, Fan AP (2019) BOLD signal physiology: models and applications. Neuroimage 187:116–127. 29544818 10.1016/j.neuroimage.2018.03.018

[CIT0025] Goñi J, van den Heuvel MP, Avena-Koenigsberger A, Velez de Mendizabal N, Betzel RF, Griffa A, Hagmann P, Corominas-Murtra B, Thiran JP, Sporns O (2014) Resting-brain functional connectivity predicted by analytic measures of network communication. Proc Natl Acad Sci U S A 111:833–838. 24379387 10.1073/pnas.1315529111 PMC3896172

[CIT0026] Griffa A, Baumann PS, Ferrari C, Do KQ, Conus P, Thiran JP, Hagmann P (2015) Characterizing the connectome in schizophrenia with diffusion spectrum imaging. Hum Brain Mapp 36:354–366. 25213204 10.1002/hbm.22633 PMC6869008

[CIT0027] Gruetter R (1993) Automatic, localized in vivo adjustment of all first- and second-order shim coils. Magn Reson Med 29:804–811. 8350724 10.1002/mrm.1910290613

[CIT0028] Hallquist MN, Hillary FG (2019) Graph theory approaches to functional network organization in brain disorders: a critique for a brave new small-world. Netw Neurosci 3:1–26. 30793071 10.1162/netn_a_00054 PMC6326733

[CIT0029] Hardingham GE, Do KQ (2016) Linking early-life NMDAR hypofunction and oxidative stress in schizophrenia pathogenesis. Nat Rev Neurosci 17:125–134. 26763624 10.1038/nrn.2015.19

[CIT0030] Honey CJ, Thivierge JP, Sporns O (2010) Can structure predict function in the human brain? Neuroimage 52:766–776. 20116438 10.1016/j.neuroimage.2010.01.071

[CIT0031] Jones DK, Christiansen KF, Chapman RJ, Aggleton JP (2013) Distinct subdivisions of the cingulum bundle revealed by diffusion MRI fibre tracking: implications for neuropsychological investigations. Neuropsychologia 51:67–78. 23178227 10.1016/j.neuropsychologia.2012.11.018 PMC3611599

[CIT0032] Karbasforoushan H, Duffy B, Blackford JU, Woodward ND (2015) Processing speed impairment in schizophrenia is mediated by white matter integrity. Psychol Med 45:109–120. 25066842 10.1017/S0033291714001111 PMC5297385

[CIT0033] Kates WR, Olszewski AK, Gnirke MH, Kikinis Z, Nelson J, Antshel KM, Fremont W, Radoeva PD, Middleton FA, Shenton ME, Coman IL (2015) White matter microstructural abnormalities of the cingulum bundle in youths with 22q11.2 deletion syndrome: associations with medication, neuropsychological function, and prodromal symptoms of psychosis. Schizophr Res 161:76–84. 25066496 10.1016/j.schres.2014.07.010 PMC4277733

[CIT0034] Klauser P, Baker ST, Cropley VL, Bousman C, Fornito A, Cocchi L, Fullerton JM, Rasser P, Schall U, Henskens F, Michie PT, Loughland C, Catts SV, Mowry B, Weickert TW, Shannon Weickert C, Carr V, Lenroot R, Pantelis C, Zalesky A (2017) White matter disruptions in schizophrenia are spatially widespread and topologically converge on brain network hubs. Schizophr Bull 43:425–435. 27535082 10.1093/schbul/sbw100 PMC5605265

[CIT0090] Klauser P, Xin L, Fournier M, Griffa A, Cleusix M, Jenni R, Cuenod M, Gruetter R, Hagmann P, Conus P, Baumann PS, Do KQ (2018) N-acetylcysteine add-on treatment leads to an improvement of fornix white matter integrity in early psychosis: a double-blind randomized placebo-controlled trial. Translational psychiatry 8:220. 10.1038/s41398-018-0266-8 PMC6185923 30315150

[CIT0035] Kochunov P, et al (2017) Association of white matter with core cognitive deficits in patients with schizophrenia. JAMA Psychiatry 74:958–966. 28768312 10.1001/jamapsychiatry.2017.2228 PMC5710230

[CIT0036] Lavoie S, Murray MM, Deppen P, Knyazeva MG, Berk M, Boulat O, Bovet P, Bush AI, Conus P, Copolov D, Fornari E, Meuli R, Solida A, Vianin P, Cuénod M, Buclin T, Do KQ (2008) Glutathione precursor, N-acetyl-cysteine, improves mismatch negativity in schizophrenia patients. Neuropsychopharmacology 33:2187–2199. 18004285 10.1038/sj.npp.1301624

[CIT0037] Li T, et al (2017) Brain-wide analysis of functional connectivity in first-episode and chronic stages of schizophrenia. Schizophr Bull 43:436–448. 27445261 10.1093/schbul/sbw099 PMC5605268

[CIT0038] Logothetis NK, Wandell BA (2004) Interpreting the BOLD signal. Annu Rev Physiol 66:735–769. 14977420 10.1146/annurev.physiol.66.082602.092845

[CIT0039] Margulies DS, Kelly AM, Uddin LQ, Biswal BB, Castellanos FX, Milham MP (2007) Mapping the functional connectivity of anterior cingulate cortex. Neuroimage 37:579–588. 17604651 10.1016/j.neuroimage.2007.05.019

[CIT0040] Mekle R, Mlynárik V, Gambarota G, Hergt M, Krueger G, Gruetter R (2009) MR spectroscopy of the human brain with enhanced signal intensity at ultrashort echo times on a clinical platform at 3T and 7T. Magn Reson Med 61:1279–1285. 19319893 10.1002/mrm.21961

[CIT0041] Monin A, Baumann PS, Griffa A, Xin L, Mekle R, Fournier M, Butticaz C, Klaey M, Cabungcal JH, Steullet P, Ferrari C, Cuenod M, Gruetter R, Thiran JP, Hagmann P, Conus P, Do KQ (2015) Glutathione deficit impairs myelin maturation: relevance for white matter integrity in schizophrenia patients. Mol Psychiatry 20:827–838. 25155877 10.1038/mp.2014.88

[CIT0042] Parente F, Frascarelli M, Mirigliani A, Di Fabio F, Biondi M, Colosimo A (2018) Negative functional brain networks. Brain Imaging Behav 12:467–476. 28353136 10.1007/s11682-017-9715-x

[CIT0043] Pettersson-Yeo W, Allen P, Benetti S, McGuire P, Mechelli A (2011) Dysconnectivity in schizophrenia: where are we now? Neurosci Biobehav Rev 35:1110–1124. 21115039 10.1016/j.neubiorev.2010.11.004

[CIT0044] Preisig M, Fenton BT, Matthey ML, Berney A, Ferrero F (1999) Diagnostic interview for genetic studies (DIGS): inter-rater and test-retest reliability of the french version. Eur Arch Psychiatry Clin Neurosci 249:174–179. 10449592 10.1007/s004060050084

[CIT0045] Provencher SW (1993) Estimation of metabolite concentrations from localized in vivo proton NMR spectra. Magn Reson Med 30:672–679. 8139448 10.1002/mrm.1910300604

[CIT0046] Rapado-Castro M, Dodd S, Bush AI, Malhi GS, Skvarc DR, On ZX, Berk M, Dean OM (2017) Cognitive effects of adjunctive N-acetyl cysteine in psychosis. Psychol Med 47:866–876. 27894373 10.1017/S0033291716002932

[CIT0047] Retsa C, Knebel JF, Geiser E, Ferrari C, Jenni R, Fournier M, Alameda L, Baumann PS, Clarke S, Conus P, Do KQ, Murray MM (2018) Treatment in early psychosis with N-acetyl-cysteine for 6months improves low-level auditory processing: pilot study. Schizophr Res 191:80–86. 28711476 10.1016/j.schres.2017.07.008

[CIT0048] Rubinov M, Sporns O (2010) Complex network measures of brain connectivity: uses and interpretations. Neuroimage 52:1059–1069. 19819337 10.1016/j.neuroimage.2009.10.003

[CIT0049] Sepehrmanesh Z, Heidary M, Akasheh N, Akbari H, Heidary M (2018) Therapeutic effect of adjunctive N-acetyl cysteine (NAC) on symptoms of chronic schizophrenia: a double-blind, randomized clinical trial. Prog Neuropsychopharmacol Biol Psychiatry 82:289–296. 29126981 10.1016/j.pnpbp.2017.11.001

[CIT0050] Shungu DC (2012) N-acetylcysteine for the treatment of glutathione deficiency and oxidative stress in schizophrenia. Biol Psychiatry 71:937–938. 22579304 10.1016/j.biopsych.2012.03.025

[CIT0051] Skåtun KC, Kaufmann T, Doan NT, Alnæs D, Córdova-Palomera A, Jönsson EG, Fatouros-Bergman H, Flyckt L, Melle I, Andreassen OA, Agartz I, Westlye LT; KaSP (2017) Consistent functional connectivity alterations in schizophrenia spectrum disorder: a multisite study. Schizophr Bull 43:914–924. 27872268 10.1093/schbul/sbw145 PMC5515107

[CIT0052] Steullet P, Cabungcal JH, Monin A, Dwir D, O’Donnell P, Cuenod M, Do KQ (2016) Redox dysregulation, neuroinflammation, and NMDA receptor hypofunction: a “central hub” in schizophrenia pathophysiology? Schizophr Res 176:41–51. 25000913 10.1016/j.schres.2014.06.021 PMC4282982

[CIT0053] van den Heuvel MP, Fornito A (2014) Brain networks in schizophrenia. Neuropsychol Rev 24:32–48. 24500505 10.1007/s11065-014-9248-7

[CIT0054] van den Heuvel MP, de Lange SC, Zalesky A, Seguin C, Yeo BTT, Schmidt R (2017) Proportional thresholding in resting-state fmri functional connectivity networks and consequences for patient-control connectome studies: issues and recommendations. Neuroimage 152:437–449. 28167349 10.1016/j.neuroimage.2017.02.005

[CIT0055] van Wijk BC, Stam CJ, Daffertshofer A (2010) Comparing brain networks of different size and connectivity density using graph theory. Plos One 5:e13701. 21060892 10.1371/journal.pone.0013701 PMC2965659

[CIT0056] Wang D, Zhou Y, Zhuo C, Qin W, Zhu J, Liu H, Xu L, Yu C (2015) Altered functional connectivity of the cingulate subregions in schizophrenia. Transl Psychiatry 5:e575. 26035059 10.1038/tp.2015.69 PMC4490280

[CIT0057] Wedeen VJ, Wang RP, Schmahmann JD, Benner T, Tseng WY, Dai G, Pandya DN, Hagmann P, D’Arceuil H, de Crespigny AJ (2008) Diffusion spectrum magnetic resonance imaging (DSI) tractography of crossing fibers. Neuroimage 41:1267–1277. 18495497 10.1016/j.neuroimage.2008.03.036

[CIT0058] Whitfield-Gabrieli S, Thermenos HW, Milanovic S, Tsuang MT, Faraone SV, McCarley RW, Shenton ME, Green AI, Nieto-Castanon A, LaViolette P, Wojcik J, Gabrieli JD, Seidman LJ (2009) Hyperactivity and hyperconnectivity of the default network in schizophrenia and in first-degree relatives of persons with schizophrenia. Proc Natl Acad Sci U S A 106:1279–1284. 19164577 10.1073/pnas.0809141106 PMC2633557

[CIT0059] Whitford TJ, Lee SW, Oh JS, de Luis-Garcia R, Savadjiev P, Alvarado JL, Westin CF, Niznikiewicz M, Nestor PG, McCarley RW, Kubicki M, Shenton ME (2014) Localized abnormalities in the cingulum bundle in patients with schizophrenia: a diffusion tensor tractography study. Neuroimage Clin 5:93–99. 25003032 10.1016/j.nicl.2014.06.003 PMC4081981

[CIT0060] Xin L, Mekle R, Fournier M, Baumann PS, Ferrari C, Alameda L, Jenni R, Lu H, Schaller B, Cuenod M, Conus P, Gruetter R, Do KQ (2016) Genetic polymorphism associated prefrontal glutathione and its coupling with brain glutamate and peripheral redox status in early psychosis. Schizophr Bull 42:1185–1196. 27069063 10.1093/schbul/sbw038 PMC4988744

[CIT0061] Yan H, Tian L, Yan J, Sun W, Liu Q, Zhang YB, Li XM, Zang YF, Zhang D (2012) Functional and anatomical connectivity abnormalities in cognitive division of anterior cingulate cortex in Schizophrenia. PLoS One 7:e45659. 23049832 10.1371/journal.pone.0045659 PMC3458074

[CIT0062] Yu C, Zhou Y, Liu Y, Jiang T, Dong H, Zhang Y, Walter M (2011) Functional segregation of the human cingulate cortex is confirmed by functional connectivity based neuroanatomical parcellation. Neuroimage 54:2571–2581. 21073967 10.1016/j.neuroimage.2010.11.018

[CIT0063] Zalesky A, Fornito A, Seal ML, Cocchi L, Westin CF, Bullmore ET, Egan GF, Pantelis C (2011) Disrupted axonal fiber connectivity in schizophrenia. Biol Psychiatry 69:80–89. 21035793 10.1016/j.biopsych.2010.08.022 PMC4881385

[CIT0064] Zalesky A, Fornito A, Cocchi L, Gollo LL, van den Heuvel MP, Breakspear M (2016) Connectome sensitivity or specificity: which is more important? Neuroimage 142:407–420. 27364472 10.1016/j.neuroimage.2016.06.035

[CIT0065] Zhang F, Qiu L, Yuan L, Ma H, Ye R, Yu F, Hu P, Dong Y, Wang K (2014) Evidence for progressive brain abnormalities in early schizophrenia: a cross-sectional structural and functional connectivity study. Schizophr Res 159:31–35. 25176348 10.1016/j.schres.2014.07.050

